# Study design and methods of the BoTULS trial: a randomised controlled trial to evaluate the clinical effect and cost effectiveness of treating upper limb spasticity due to stroke with botulinum toxin type A

**DOI:** 10.1186/1745-6215-9-59

**Published:** 2008-10-23

**Authors:** Helen Rodgers, Lisa Shaw, Christopher Price, Frederike van Wijck, Michael Barnes, Laura Graham, Gary Ford, Phil Shackley, Nick Steen, BoTULS investigators

**Affiliations:** 1 Institute for Ageing and Health (Stroke Research Group), Newcastle University, Newcastle upon Tyne NE4 5PL, UK; 2 Institute of Health and Society, Newcastle University, Newcastle Upon Tyne NE2 4AA, UK; 3 Northumbria Healthcare NHS Trust, Wansbeck General Hospital, Ashington, Northumberland NE63 9JJ, UK; 4 School of Health Sciences, Queen Margaret University, Queen Margaret University Drive, Edinburgh EH21 6UU, UK; 5 International Centre for Neurorehabilitation, Walkergate Park, Benfield Road, Newcastle upon Tyne NE6 4QD, UK; 6 School of Medicine and Biomedical Sciences, University of Sheffield, Samuel Fox House, Northern General Hospital, Sheffield S5 7AU, UK

## Abstract

**Background:**

Following a stroke, 55–75% of patients experience upper limb problems in the longer term. Upper limb spasticity may cause pain, deformity and reduced function, affecting mood and independence. Botulinum toxin is used increasingly to treat focal spasticity, but its impact on upper limb function after stroke is unclear.

The aim of this study is to evaluate the clinical and cost effectiveness of botulinum toxin type A plus an upper limb therapy programme in the treatment of post stroke upper limb spasticity.

**Methods:**

Trial design : A multi-centre open label parallel group randomised controlled trial and economic evaluation.

Participants : Adults with upper limb spasticity at the shoulder, elbow, wrist or hand and reduced upper limb function due to stroke more than 1 month previously.

Interventions : Botulinum toxin type A plus upper limb therapy (intervention group) or upper limb therapy alone (control group).

Outcomes : Outcome assessments are undertaken at 1, 3 and 12 months. The primary outcome is upper limb function one month after study entry measured by the Action Research Arm Test (ARAT). Secondary outcomes include: spasticity (Modified Ashworth Scale); grip strength; dexterity (Nine Hole Peg Test); disability (Barthel Activities of Daily Living Index); quality of life (Stroke Impact Scale, Euroqol EQ-5D) and attainment of patient-selected goals (Canadian Occupational Performance Measure). Health and social services resource use, adverse events, use of other antispasticity treatments and patient views on the treatment will be compared. Participants are clinically reassessed at 3, 6 and 9 months to determine the need for repeat botulinum toxin type A and/or therapy.

Randomisation : A web based central independent randomisation service.

Blinding : Outcome assessments are undertaken by an assessor who is blinded to the randomisation group.

Sample size : 332 participants provide 80% power to detect a 15% difference in treatment successes between intervention and control groups. Treatment success is defined as improvement of 3 points for those with a baseline ARAT of 0–3 and 6 points for those with ARAT of 4–56.

**Trial registration:**

ISRCTN78533119

EudraCT 2004-002427-40

CTA 17136/0230/001

**Funding:**

National Institute for Health Research, Health Technology Assessment Programme.

Ipsen Ltd provide botulinum toxin type A (Dysport^®^).

## Background

### Upper limb spasticity following stroke

Upper limb impairment affects 85% of stroke patients, many of whom still experience problems in the longer term[1,2]. Spasticity can occur following stroke and is challenging to define and measure. Most recently it has been described as "disordered sensori-motor control, resulting from an upper motor neurone lesion, presenting as intermittent or sustained involuntary activation of muscles"[3]. An older, narrower and more commonly quoted definition is " a motor disorder characterised by a velocity dependent increase in tonic stretch reflexes with exaggerated tendon reflexes, resulting from hyperexcitability of the stretch reflex"[4]. However, put simply spasticity is over activity of muscles as a result of damage to the brain or spinal cord. Spasticity can cause pain and deformity, and in the longer term may lead to the development of contractures[5,6]. Upper limb spasticity can lead to reduced arm function and problems with ease of hygiene[6]. Effective management of spasticity requires a coordinated multidisciplinary team approach[6].

### Randomised controlled trials of botulinum toxin in the treatment of upper limb spasticity following stroke

After injection botulinum toxin causes local paresis of muscles as a result of blocking cholinergic transmission at the neuromuscular junction. The clinical treatment effect lasts for 3–4 months[6]. To date, nine randomised controlled trials have evaluated its use for the treatment of upper limb spasticity following stroke [7-15], and three systematic reviews have been published [16-18] (two trials[14,15] and two systematic reviews were published following the start of this study[17,18]).

Trials have reported a measurable reduction in resistance to passive movement on the Modified Ashworth Scale within 6 weeks, which then reduces towards 12–16 weeks, often losing statistical significance at this time. The main benefits of spasticity reduction appear to be in terms of global patient/physician ratings and itemised passive disability scores (notably hand hygiene). Only one study (published after this study commenced) has reported an improvement in active upper limb function[15].

As the treatment effect of botulinum toxin lasts only 3–4 months, injections need to be repeated to offer sustained benefit. Only one trial (published after this study commenced) has considered the impact of repeat injections[14]. This has provided limited evidence to support continued use of botulinum toxin for spasticity reduction.

Guidelines for the use of botulinum toxin in the treatment of spasticity recommend that it should be used in combination with a rehabilitation programme[6], but no trial to date has attempted to standardise upper limb therapy. No study has looked at the cost-effectiveness of treatment. Although transient muscle weakness at higher doses of botulinum toxin is well recognised, studies reported no unexpected adverse events, however, the event reporting system was often unclear.

Participants in previous studies were significantly younger (average age 52–66 years) than typical stroke patients (the average incident age of stroke is 75 years) and the studies were often undertaken in specialist rehabilitation centres. Multidisciplinary care on a stroke unit is currently the gold standard for stroke rehabilitation and benefits are seen regardless of age or stroke severity[19]. Evaluations of botulinum toxin should recruit participants of all ages from stroke services rather than tertiary referral centres to avoid selection bias and ensure results are applicable to routine care.

The aim of this study is to evaluate the clinical and cost effectiveness of botulinum toxin type A plus an upper limb therapy programme in the treatment of post stroke upper limb spasticity.

## Methods

### Study design

**Figure 1:**
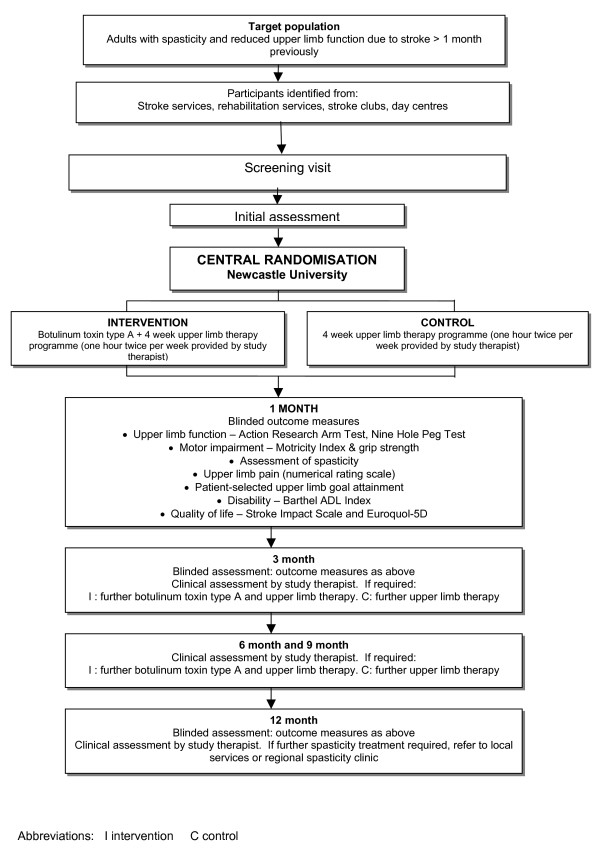
Study method.

This is a multi-centre open-label parallel group randomised controlled trial comparing the clinical and cost effectiveness of botulinum toxin type A plus an upper limb therapy programme with the upper limb therapy programme alone for the treatment of upper limb spasticity due to stroke in adults. Figure 1 outlines the study method.

### Primary objective

1. To compare the upper limb function of participants with spasticity due to stroke who receive botulinum toxin type A injection(s) to the upper arm and/or forearm flexors/hand/shoulder girdle plus a four week evidence based upper limb therapy programme (intervention group) with participants who receive the upper limb therapy programme alone (control group) one month after study entry. Upper limb function is assessed using the Action Research Arm Test (ARAT)[20].

### Secondary objectives

1. To compare the upper limb function and impairment of participants with spasticity due to stroke who receive botulinum toxin type A injection(s) to the upper arm and/or forearm flexors/hand/shoulder girdle plus a four week evidence based upper limb therapy programme (intervention group) with participants who receive the upper limb therapy programme (control group) 1, 3 and 12 months after study entry. Upper limb function and impairment is assessed by: ARAT[20], Motricity Index[21], grip strength[22], nine hole peg test[23], and Modified Ashworth scale[24].

2. To compare attainment of participant-selected upper limb goals, disability and stroke related quality of life between intervention and control groups at 1,3 and 12 months. The following measures are used: attainment of participant-selected upper limb goals (1 month only) – Canadian Occupational Performance Measure (COPM)[25]; disability – Barthel ADL Index[26]; quality of life – Stroke Impact Scale[27], Euroqol EQ-5D[28], numerical rating scales for upper limb pain[29].

3. To seek the experience and views of participants about treatment at 1 and 12 months.

4. To compare the health and social services resources used by control and intervention groups during the 12 months following study entry.

5. To report adverse events and compare the use of other antispasticity treatments between intervention and control groups.

6. To investigate the influence of severity of upper limb impairment and time since stroke upon the efficacy of the intervention.

### Setting

The study involves a collaborative network of twelve stroke services in the north of England, with expertise in the management of spasticity and use of botulinum toxin being provided by the International Centre for Neurorehabilitation, Newcastle. We believe that the model which we have developed i.e. stroke units with close links to a specialist spasticity service enables all stroke patients to access specialist care (both in terms of stroke and spasticity management) and this model could be replicated in other settings.

### Case ascertainment

1. Potential participants are identified from a number of sources in each study centre (stroke unit, out-patients, day hospital and community rehabilitation teams). He/she is given an information leaflet and has an opportunity to discuss the study with a member of the clinical team (training is given to clinical teams about the project and research governance). The research team then arranges to see him/her to discuss the study and seek consent at a screening visit.

2. There are potential participants who are not currently in contact with rehabilitation or stroke services. Local community stroke clubs and day centres have been given information about the study and individuals may contact the study directly.

### Inclusion criteria

Adults with a stroke greater than 1 month previously who have moderate/severe spasticity and reduced upper limb function who fulfil all of following criteria are eligible:

▪ Age over 18 years.

▪ At least 1 month since stroke.

▪ Upper limb spasticity (Modified Ashworth Scale[24] > 2 at the elbow and/or spasticity at the hand, wrist, or shoulder (there is no validated measure of spasticity at these sites)).

▪ Reduced upper limb function (ARAT[20] score 0–56)

▪ Able to comply with the requirements of the protocol and upper limb therapy programme.

▪ Informed consent given by participant or legal representative.

### Exclusion criteria

▪ Significant speech or cognitive impairment which impedes ability to perform the ARAT[20] assessment.

▪ Other significant upper limb impairment e.g. fracture or frozen shoulder within six months, severe arthritis, amputation.

▪ Evidence of fixed contracture.

▪ Pregnancy or lactating.

▪ Female at risk of pregnancy and not willing to take adequate precautions against pregnancy for the duration of the study.

▪ Other diagnosis likely to interfere with rehabilitation or outcome assessments e.g. registered blind, malignancy.

▪ Other diagnosis which may contribute to upper limb spasticity e.g. multiple sclerosis, cerebral palsy.

▪ Contraindications to intramuscular injection.

▪ Religious objections to blood products (botulinum toxin type A (Dysport^®^) contains human albumin).

▪ Contraindications to botulinum toxin type A which include bleeding disorders, myasthenia gravis and concurrent use of aminoglycosides.

▪ Use of botulinum toxin to the upper limb in the previous three months.

▪ Known allergy or hypersensitivity to any of the test compounds.

▪ Previous enrolment in this study.

### Screening visit

Having sought consent, the screening assessment is completed by a study therapist or clinical research associate. The assessment consists of demographic details, review of medical history and medication; handedness; Abbreviated Mental Test Score[30], Sheffield Aphasia Screening Test[31], pre-stroke function (Oxford Handicap Scale)[32]; time since stroke; stroke type and subtype [33]; self reported current neurological impairment and function (Barthel ADL Index[26]); quality of life (Euroqol EQ-5D[28]). Details of current and anti-spasticity treatment received within the previous 3 months and concomitant medications are recorded.

### Baseline assessment

The baseline visit is undertaken within 2 weeks of the screening visit by a study therapist or clinical research associate. The inclusion/exclusion criteria are reviewed to ensure that the participant is still eligible. Participants undergo a clinical assessment and are asked to complete a battery of assessments including: Action Research Arm Test[20]; Motricity Index[21]; grip strength[22]; Nine Hole Peg Test[23]; Modified Ashworth Scale[24]; upper limb pain[29] and Stroke Impact Scale[27]. Patient selected upper limb goals are also identified (Canadian Occupational Performance Measure)[25]. Female participants of child-bearing potential (i.e. those who are not either surgically sterile or at least 1 year post-last menstrual period) have a urine pregnancy test, the result of which must be negative for the participant to be included in the study. Such participants must agree to use adequate contraception throughout the study if they are randomised to receive botulinum toxin type A. Participants are randomised once the baseline assessment has been completed.

### Randomisation

Randomisation is by a central independent web based randomisation service from the Clinical Trials Unit, Newcastle University. Participants are stratified according to level of upper limb function (ARAT 0–3, ARAT 4–28, ARAT 29–56) and randomised to intervention or control in a 1:1 ratio.

### Botulinum toxin

Participants in the intervention group receive botulinum toxin type A (Dysport^®^). The range of muscles and dosages injected are described in 'The management of adults with spasticity using botulinum toxin: a guide to clinical practice'[6]. The maximum dose of botulinum toxin type A (Dysport^®^) administered at any one time point is 1000 units. All injectors are clinicians trained in the assessment and injection of botulinum toxin in the context of upper limb spasticity.

If further treatment is necessary at three, six or nine months a further injection is provided to those in the intervention group. At the 12-month review individuals in both the intervention and control groups who require botulinum toxin are referred to the spasticity clinic.

If during the course of the trial the study therapist decides that a participant in the control group has an unacceptable degree of symptomatic spasticity he/she discusses further management with their stroke physician, physiotherapist, occupational therapist and/or a member of the local or regional spasticity team and the participant may then be referred to the spasticity service for botulinum toxin.

### The upper limb rehabilitation programme

Guidelines highlight that it is important that botulinum toxin is not used in isolation but as part of a comprehensive upper limb therapy programme[5,6,34]. Focal reductions in upper limb spasticity from any pharmacological intervention are unlikely to translate into sustained improvements in function or patient-selected rehabilitation goals without a targeted therapy programme.

The upper limb therapy programme is based upon available research evidence from the stroke rehabilitation and skill acquisition literature as well as clinical practice [34-52], and consists of two menus. Participants with ARAT 0–3 receive menu 1 which is designed specifically for participants with no active hand function and focuses on stretching, passive and active-assisted upper limb movement along with hand hygiene and positioning [42-47]. Menu 2 is for participants with some retained active upper limb movement (ARAT 4–56) and has been piloted in a previous study[53]. Following stretching of soft tissues affected by spasticity, this menu specifically concentrates on intensive task-orientated practice aimed at patient-centred goals. Upper limb goals are measured by the Canadian Occupational Performance Measure[25]. Each menu standardises the category of tasks, the number and order of repetitions as well as the amount of feedback for each session, but within these parameters the therapist can tailor the specifics of each activity to the ability of the patient. Manuals and training programmes have been developed for both upper limb therapy menus.

The upper limb therapy programme is provided by study therapists and each participant receives one hour per day, two times per week for four weeks, in addition to their other rehabilitation needs. The study therapist may transfer participants between menu 1 and menu 2 according to their clinical opinion. Participants are given a written exercise programme to carry out by themselves or with a carer (following training) on the weekdays they are not attending therapy.

If the participant is currently receiving rehabilitation, then the upper limb therapy programme is delivered in that setting e.g. stroke unit, out-patients, day hospital or home. In each case, the study therapist liaises closely with the rehabilitation team to ensure the participant's needs are addressed and well co-ordinated. At the end of the four week intervention period patients are given advice by the study therapist regarding maintaining upper limb function.

Participants are reviewed by the research team every three months. If further therapy is required, this is provided by a study therapist. Those in the intervention group may also receive a further botulinum toxin type A injection. Participants in both the intervention and control group who have symptomatic spasticity at the 12 month follow up appointment are referred to a spasticity clinic.

Participants who make a good recovery prior to completing the four week upper limb therapy programme are discharged from the programme provided that they have a maximum score on the ARAT[20] and have achieved their upper limb goals.

### Outcome assessments

Outcomes are measured by an assessor who is blinded to the randomisation group one month (+/- 3 days), three months (+/- 5 days) and twelve months (+/- 5 days) after the baseline visit. Before contacting the participant a member of the research team checks with their general practice or stroke unit that they are still alive and checks their address. The medical records of participants who have died are reviewed to seek details of the cause and circumstances of death, resource utilisation data, and any potential side effects from botulinum toxin. Each outcome assessment consists of two stages – stage 1 outcome assessment by a self completion postal questionnaire (Barthel ADL Index[26], quality of life (Stroke Impact Scale[27], Euroqol EQ-5D[28], resource utilisation) which is sent to participants one week prior to stage 2. Participants are asked to bring the completed proforma to their stage 2 appointment.

Stage 2 outcome assessments consists of assessment of upper limb impairment and function (ARAT[20], Motricity Index[21], grip strength[22], Nine Hole Peg Test[23], assessment of spasticity[24] and upper limb pain[29]) and face to face interview seeking participants experience and views of the study treatment. Information is sought about side effects, use of other antispasticity treatment and analgesia for post stroke upper limb pain. Any change in the participant's concomitant medications since the previous visit is noted. Any new adverse events or changes in existing adverse events that have occurred since the previous visit are sought. The stage 1 questionnaire is checked for completeness.

### Blinding

Outcome assessments are undertaken by an assessor who is blinded to the randomisation group. To enable blinding to be achieved study therapists undertake screening and baseline assessments and provide the upper limb therapy programme in one research centre and undertake outcome assessments in adjacent centres. At each outcome assessment he/she is asked to record if he or she had become unblinded. Participants and the study therapists who provide the upper limb therapy programme are not blind to the randomisation group.

### Safety evaluation

Side effects of botulinum toxin type A are generally mild and transient. Local muscle weakness may occur as a result of toxin spread to nearby muscles. Five per cent experience flu like symptoms 1 week to 10 days post injection, dry mouth and pain at the injection site can occur. Transient dysphagia has been reported. Anaphylaxis rarely occurs. Excessive doses may produce distant and profound neuromuscular paralysis. Respiratory support may be required where excessive doses cause paralysis of respiratory muscles. The safety of botulinum toxin type A in the treatment of participants with upper limb spasticity post stroke is evaluated by examining the occurrence of all adverse and serious adverse events as defined by the EU clinical trial directive[54]. Follow-up of each adverse event continues until the event or its sequelae resolve or stabilise at a level that is acceptable to the investigator.

### Resource utilisation and economic evaluation

Health and social service resource use associated with botulinum toxin type A therapy is costed according to established methods [55-58]. Measurement and valuation of resource use is undertaken using a combination of routine administrative data and primary data collection methods devised specifically for the study. Costs measured and valued include the costs of the drugs, costs of the upper limb rehabilitation programme (e.g. staff time) and costs associated with any adverse side effects associated with the administration of the drugs (e.g. general practitioner or other health care contacts, contacts with social services, out of pocket payments by participants for pain relieving drugs). The costs of the drugs is ascertained from suppliers. Staff time is recorded and costed using gross hourly wage rates derived from the mid-point of appropriate salary scales. Costs of rehabilitation equipment e.g. upper limb splinting is obtained from routine data. The frequency of participant contacts with health professionals and social services is ascertained through the administration of a participant questionnaire at the 1, 3 and 12 month outcome assessments. Participants are asked about any contacts they have had with health care professionals and social services since their last assessment and to indicate any out of pocket expenditure they have incurred for medication. As the study period is 12 months, there is no need to apply discounting. The economic evaluation combines the cost data with data on outcomes from the Euroqol EQ-5D[28] which is used to estimate the quality adjusted life years (QALYS) gained from the intervention. An incremental cost per QALY gained for botulinum toxin plus upper limb therapy relative to therapy alone will be estimated and associated cost-effectiveness acceptability curves presented. Sensitivity analysis will be undertaken to assess the sensitivity of the results to variations in key parameters.

### Study schedule

Table 1 summarises the study schedule.

**Table 1:** Study schedule

Time point	**Screening** < 2 weeks	**Baseline** Day 0	**Visit 3** Month 1^1^	**Visit 4** Month 3^2^	**Visit 5** Month 6	**Visit 6** Month 9	**Visit 7** Month 12^2^
Informed consent	X						
Record demographics & handedness	X						
Review inclusion/exclusion criteria	X	X					
Review medical history	X	X					
Details of stroke	X						
Pre stroke function (inc Oxford Handicap Scale^)^)	X						
Abbreviated Mental Test Score (AMTS)	X						
Sheffield Aphasia Screening Test	X						
Action Research Arm Test (ARAT)	X	X	X	X			X
Motricity Index		X	X	X			X
Grip strength		X	X	X			X
Nine Hole Peg Test		X	X	X			X
Modified Ashworth Scale	X	X	X	X			X
Self rating of severity		X	X	X			X
Upper limb pain (numerical rating scale)^3^		X	X	X			X
Patient selects upper limb goals		X					
Review upper limb goal attainment			X				
Barthel ADL Index^3^	X		X^3^	X^3^			X^3^
Quality of life – Stroke Impact Scale^3^	X		X^3^	X^3^			X^3^
Quality of life – Euroquol-5D^3^	X		X^3^	X^3^			X^3^
Resource utilisation questions^3^	X		X^3^	X^3^			X^3^
Pregnancy test^4^		X		X^6^	X^6^	X^6^	
Randomisation		X					
Treatment with Dysport^5^		X		X^7^	X^7^	X^7^	
Commencement of 4 week upper limb therapy programme		X		X^7^	X^7^	X^7^	
Clinical assessment by study therapist		X		X	X	X	X
Concomitant medications (inc anti-spasticity treatment)	X	X	X	X			X
Adverse Events		X	X	X			X
Participants views and experience			X				X

1. Visit window is +3 days.

2. Visit window is +5 days.

3. Questionnaires will be sent to the participant for completion 1 week prior to the visit. Participants will bring completed forms to the visit.

4. For female participants at risk of pregnancy.

5. Participants in the intervention group only.

6. Pregnancy test to be performed prior to any additional botulinum toxin injections.

7. Additional botulinum toxin injections/upper limb therapy to be provided if clinically appropriate

### Statistical analysis

The primary endpoint is the ARAT[20] score at 1 month. The analysis will be undertaken on an "intention to treat" basis; participants will analysed in the group to which they were randomised.

For each participant it will be determined if there has been a significant improvement in function based on the change in ARAT score. A successful outcome will be defined as:

(i) a change of 3 or more points on the ARAT scale for a participant whose baseline ARAT score is between 0 and 3

(ii) a change of 6 or more points on the ARAT scale for a participant whose baseline ARAT score is between 4 and 51

(iii) a final ARAT score of 57 for a participant whose baseline ARAT score is 52 or greater.

The proportion of "successes" in each group will then be compared using Fisher's exact test. An interval estimate of the relative odds of a successful outcome in each group will also be calculated. This interval estimate of effect size will be taken forward into the economic evaluation.

Secondary outcomes will also be analysed on an "intention to treat" basis. A test appropriate to the type of variable will be undertaken.

A pre-specified sub group analysis will consider the effect of time since stroke as a covariate. It is hypothesised that participants who have recently had a stroke will have a better response to treatment than those who had a stroke some time ago.

The power calculation used prognosis based methodology[59]. A clinically important treatment effect is defined as a difference in good outcomes between intervention and control groups of 15% where a good outcome is defined above for each of the ARAT group; it is expected to see 20% of the control group achieve good outcomes and 35% of the intervention group achieve good outcomes. Using Fleiss' method[60] for a binary outcome and inflating the sample size by 10% to allow for attrition we need to recruit a total sample of 332 participants to give us 80% power to detect a 15% difference in good outcomes assuming a two-tailed test and a significance level of 5%. We are aiming to recruit 50% of the sample from the ARAT 0–3 group and 50% from the ARAT 4–56 group.

### Current study status

The study commenced recruitment in July 2005 and achieved target recruitment in March 2008. Participant follow up was completed in June 2008. The results of the study will be submitted for publication end 2008.

### Amendments to the study since commencement (July 2005)

#### (1) Objectives

The study protocol includes measurement of spasticity at the elbow by a biomechanical device which has been used in a previous pilot study[61]. This was to be used in addition to clinical measures. Unfortunately the device is not at a stage of development where it can be used in a multicentre study.

#### (2) Setting

Initially the trial was planned in 4 geographical areas: North Tyneside, Wansbeck, Newcastle and Sunderland. Due to low recruitment rates, further sites were added.

#### (3) Case ascertainment

This was widened to include identification of participants from stroke clubs and day centres (in addition to clinical settings) due to initial low recruitment rates.

#### (4) Inclusion criteria

Prospective studies of upper limb recovery have shown that baseline impairment is a strong predictor of outcome. To demonstrate whether botulinum toxin plus upper limb therapy can improve arm function (primary outcome), it was initially thought important to exclude those participants with no retained upper limb function (ARAT 0–3). Due to initial low recruitment, this was later reconsidered and it was decided that it would be valuable to include stroke patients with all levels of reduced upper limb function (ARAT 0–56).

The study also initially excluded participants with cognitive impairment or significant speech problems measured by the Abbreviated Mental Test Score and Sheffield Aphasia Screening Test. This was felt to be too restrictive, excluding patients who were keen to participate. This inclusion criteria was relaxed to include all participants capable of performing the ARAT and complying with the upper limb therapy programme.

#### (5) Upper limb therapy programme

A second menu was developed for the upper limb therapy programme after the eligibility criteria were widened to include participants with no active upper limb function. This alternative menu was designed because the original menu contained activities which these participants would not have been able to undertake.

#### (6) Statistical analysis

Inclusion of participants with lower ARAT scores required revision of the primary analysis and power calculation. Expert opinion concluded that participants with a baseline ARAT of 0–3 could not be predicted to improve as much as those with a baseline ARAT of 4–56. This led to the definition of successful treatment as improvement by 3 points on the ARAT for those with a starting ARAT of 0–3 and 6 points by those with a starting ARAT of 4–56. Comparison of proportions of successes between the groups (control/intervention) became the primary analysis (as opposed to comparison of absolute ARAT scores in the initial protocol).

The power calculation was revised for the new binary outcome.

#### (7) Follow up period

Participants recruited after 2^nd ^July 2007 were followed for 3 months only. This was a pragmatic decision taken due to the trial being behind schedule from initial low recruitment rates. Curtailing 12 month follow up allows the trial to be completed within the initial study timetable. Twelve month follow up has occurred for 208/333 (62%) participants.

### Study acronym

BoTULS: Botulinum Toxin for the Upper Limb after Stroke

### Ethical arrangements and research governance

The study has MREC, MHRA and local site R+D approvals, and is being conducted in accordance with ICH-GCP[62], the UK Medicines for Human Use (Clinical Trials) Regulations 2004[54] and the Research Governance Framework for Health and Social Care[63].

The study has a Data Monitoring and Ethics Committee (Professor Martin Dennis (chair), University of Edinburgh; Professor Ian Ford, University of Glasgow; Professor Marion Walker, University of Nottingham; Dr Nick Steen, Newcastle University) and a Trial Steering Committee (Professor Michael Barnes, Newcastle University; Professor Bipin Bhakta, University of Leeds; Mrs Beryl Fairless, lay member, Newcastle upon Tyne; Professor Gary Ford, Newcastle University; Professor Anne Forster, University of Leeds; Professor Peter Langhorne (chair), University of Glasgow; Professor Helen Rodgers, Newcastle University).

The study sponsor is Newcastle Upon Tyne NHS Foundation Trust.

The study is adopted by the UK Stroke Research Network.

## Competing interests

Ipsen Ltd are providing the botulinum toxin type A used by the study free of charge. They have also provided funding for sandwich lunches at launch meetings at study sites. The design, analysis and reporting of the study is being undertaken independently of Ipsen Ltd. HR is the deputy director of the UK Stroke Research Network. LS has no competing interests. CP is the deputy director of the North East Stroke Research Network. FvW has no competing interests. MB is a consultant in rehabilitation medicine and uses botulinum toxin regularly in clinical practice. He has received sponsorship from Ipsen Ltd to attend and teach at conferences and meetings but has no personal financial interest in botulinum toxin or any related product. LG is a consultant in rehabilitation medicine and uses botulinum toxin regularly in clinical practice. She has received sponsorship from Ipsen Ltd to attend and teach at conferences and meetings but has no personal financial interest in botulinum toxin or any related product. GF is director of the UK Stroke Research Network. PS has no competing interests. NS has no competing interests.

## Authors' contributions

HR, CP, FvW, MB, GF designed the RCT. HR is the chief investigator. HR and LS drafted the paper. LS and CP undertook the literature review of previous trials. LS is the clinical research associate with responsibility for the day to day running of the study and delivery of botulinum toxin type A injections. FvW designed the upper limb therapy programme. She trained the research therapists to deliver the programme and administer the upper limb outcome measures. MB and LG provide expertise in the management of spasticity and use of botulinum toxin. LG has also been involved in the delivery of trial botulinum toxin type A injections. PS designed and is responsible for the health economics component of the study. NS is the study statistician. All authors have commented upon drafts of the paper and have given final approval to this version.
